# Frailty and medical financial hardship among older adults with cancer in the United States

**DOI:** 10.3389/fonc.2023.1202575

**Published:** 2023-06-29

**Authors:** Jiahui Lao, Mingzhu Su, Jiajun Zhang, Li Liu, Shengyu Zhou, Nengliang Yao

**Affiliations:** ^1^ Department of Oncology, The First Affiliated Hospital of Shandong First Medical University and Shandong Provincial Qianfoshan Hospital, Shandong Lung Cancer Institute, Jinan, Shandong, China; ^2^ Center for Big Data Research in Health and Medicine, The First Affiliated Hospital of Shandong First Medical University and Shandong Provincial Qianfoshan Hospital, Jinan, Shandong, China; ^3^ Centre for Health Management and Policy Research, School of Public Health, Cheeloo College of Medicine, Shandong University, Jinan, Shandong, China; ^4^ National Health Commission Key Lab of Health Economics and Policy Research (Shandong University), Jinan, Shandong, China; ^5^ School of Nursing and Rehabilitation, Cheeloo College of Medicine, Shandong University, Jinan, Shandong, China; ^6^ Home Centered Care Institute, Schaumburg, IL, United States

**Keywords:** frailty, medical financial hardship, cancer survivor, older adults, national sample

## Abstract

**Background:**

Little is known about the association between frailty level and medical financial hardship among older adults with cancer. This study aims to describe the prevalence of frailty and to identify its association with medical financial hardship among older cancer survivors in the United States.

**Methods:**

The National Health Interview Survey (NHIS; 2019–2020) was used to identify older cancer survivors (n = 3,919). Both the five-item (Fatigue, Resistance, Ambulation, Illnesses, and Low weight-for-height) FRAIL and the three-domain (Material, Psychological, and Behavioral) medical financial hardship questions were constructed based on the NHIS questionnaire. Multivariable logistic models were used to identify the frailty level associated with financial hardship and its intensity.

**Results:**

A total of 1,583 (40.3%) older individuals with cancer were robust, 1,421 (35.9%) were pre-frail, and 915 (23.8%) were frail. Compared with robust cancer survivors in adjusted analyses, frail cancer survivors were more likely to report issues with material domain (odds ratio (OR) = 3.19, 95%CI: 2.16–4.69; p < 0.001), psychological domain (OR = 1.47, 95%CI: 1.15–1.88; p < 0.001), or behavioral domain (ORs ranged from 2.19 to 2.90, all with p < 0.050), and greater intensities of financial hardship.

**Conclusion:**

Both pre-frail and frailty statuses are common in the elderly cancer survivor population, and frail cancer survivors are vulnerable to three-domain financial hardships as compared with robust cancer survivors. Ongoing attention to frailty highlights the healthy aging of older survivors, and efforts to targeted interventions should address geriatric vulnerabilities during cancer survivorship.

## 1 Introduction

Cancer is a chronic disease of aging; approximately 70% of all cancers occur in people aged ≥65 years, and the number of people with rapid growth will increase in the future (1–3). Despite more than 90% of the senior population having Medicare coverage, the high medical costs of treatment after a cancer diagnosis impose substantial financial hardship on older cancer survivors (4, 5). There is growing interest in understanding cancer-related financial hardships, needs, and sacrifices and identification of the detrimental characteristics among older individuals with cancer (5–11). Although much of this knowledge has been identified from previous studies in older cancer survivorship research areas, there is less evidence to assess the relationship between age-associated conditions and the financial hardships of medical care for this growing population. Therefore, understanding financial hardship among this population is challenging.

Age alone does not properly characterize physiological heterogeneity (12, 13). For older cancer survivors, the “stage of aging” is as important as the “stage of cancer”. Previous studies showed that the frailty status, which is emerging as one of the most important determinants of health and health outcomes, could be an ideal tool to stage aging rather than age groups among older adults (14, 15). Frailty, an age-related clinical syndrome characterized by vulnerability to stressors, has been suggested as a framework for understanding the highly individualized process of aging (16). In addition, frailty status had proven to be changeable and reversible (17). Frailty among cancer survivors is associated with increased risk for adverse events, including hospitalization, new onset of chronic disease, and mortality (18). A cohort of older community-dwelling individuals with frailty was associated with higher subsequent total direct healthcare costs after accounting for demographics, multimorbidity, cognition, and functional limitations (19). Previous studies have shown that frailty was associated with a high risk of healthcare utilization, long-term functional outcomes, and prolonged hospital stays in older adults with cancer (20–22). Additional research is warranted to determine if frailty assessment in a large population is feasible and will alleviate financial hardship regarding healthcare utilization aimed at reducing subsequent healthcare burdens. Understudied frail older adults in cancer survivorship research, especially about medical financial hardship, may hinder progress in aging-tailored interventions and strategies to effectively mitigate the financial burdens of cancer care. As a potentially modifiable age-related characteristic, frailty status could be an important intervention lever for addressing medical financial issues among older cancer survivors. To date, the study on the associations between complex frailty status and medical financial hardship of older cancer survivors is still limited.

This study used a large nationally representative sample to calculate frailty score, then quantified the prevalence of frailty in older cancer survivors, and finally compared medical financial hardship across the material, psychological, and behavioral domains stratified by frailty level. Furthermore, researchers comprehensively evaluated the association of the frailty of older cancer survivors with medical financial hardship and its intensity. Findings from this study will provide critical information needed for an understanding of health disparities and medical financial hardship in older adults with cancer, as well as key information for policymakers to have an insight into the rapidly aging population and cancer demographic of the country. Highlighting frailty status will also close the knowledge gap on aging-related consequences of cancer to enhance healthy aging among older adults with cancer.

## 2 Materials and methods

### 2.1 Data sources

The National Health Interview Survey (NHIS) data were used to identify older adults with cancer (aged ≥65 years). The NHIS is an annual, nationally representative household survey of the United States civilian non-institutionalized population. In the NHIS, cancer survivors were defined as those who reported that they had ever been told by a physician or other health professional that they had cancer or a malignancy of any kind. Due to the availability of measures on the FRAIL questionnaire, this study sample was restricted to the years 2019 and 2020. The COVID-19 pandemic impacted NHIS interviewing procedures beginning in late March 2020, so NHIS shifted from in-person interviews to all-telephone interviews starting in late March and continuing through June. Approximately one-third of the sample adult interviews in 2020 (n = 10,415) are composed of sample adults previously interviewed for the 2019 NHIS. Researchers combined the 2019 and 2020 data (excluding the 2019–2020 longitudinal sample from 2020), and the household response rate was 56.5% (23). **Supplementary Figure S1** shows the flowchart for the inclusion and exclusion of NHIS participants, and the final analysis included a sample of 3,919 cancer survivors.

### 2.2 Individual-level characteristics

Demographic characteristics included age at the time of the survey, sex, race/ethnicity, education, marital status, health insurance, family income level as a percentage of the federal poverty level (FPL), and geographic region. Cancer-related variables included the number of cancer diagnoses to define single and multiple cancers (1 cancer *vs.* ≥ 2 cancers) and time since diagnosis, which was calculated using age at most recent diagnosis and age at the survey (<2 *vs.* ≥2 years).

### 2.3 Frailty status

The FRAIL Scale, developed by the Geriatric Advisory Panel of the International Society for Nutrition and Aging, is a validated screening tool (24, 25). For this study, NHIS 2019 and 2020 data were used to construct the modified FRAIL Scale, and the FRAIL questionnaire consisted of five components: Fatigue, Resistance, Ambulation, Illness, and Low body mass index (BMI) (26). Fatigue in the 2019 NHIS was measured by asking respondents, “Over the last two weeks, how often have you been bothered by feeling tired or having little energy?” with responses of “nearly every day” or “more than half the days” scoring 1 and “not at all” or “several days” scoring as 0. Fatigue in the 2020 NHIS was measured by asking respondents, “Thinking about the last time you felt very tired or exhausted, how long did it last?” with responses of “all of the day” or “most of the day” scoring 1 and “some of the day” scoring as 0. Resistance was assessed by asking respondents, “Do you have difficulty walking up or down 12 steps without any equipment or receiving help?”, and Ambulation by asking, “Do you have difficulty walking 100 yards on level ground, that would be about the length of one football field or one city block, without any equipment or receiving help?”; “no difficulty” responses were each scored 0, and all other responses were scored 1. Illness was scored 1 for respondents who reported five or more illnesses out of 14 total illnesses (angina, anxiety disorder, arthritis, asthma, cancer, chronic obstructive pulmonary disease, coronary heart disease, dementia, depression, diabetes, heart attack, high cholesterol, hypertension, and stroke), and respondents with zero to four reported illnesses were scored 0. Low BMI was scored 1 for respondents with BMI < 18.5 kg/m^2^; otherwise, it was scored 0. Frail Scale scores ranged from 0 to 5 and represented frailty status (3–5), pre-frailty status (1, 2), and robust status (0) (25).

### 2.4 Medical financial hardship

Material financial hardship was defined as “participants or their family members having reported problems paying for medical bills in the past 12 months, or reporting any current medical bills they are unable to pay at all (only participants who had reported problems paying for medications were asked these questions)” (27, 28). Psychological medical hardship was defined as “participants having reported sickness or an accident, and are worried about being unable to pay your medical bills at the time of the survey”; this was then dichotomized into hardship (“very worried” or “somewhat worried”) or no hardship (“not worried at all”) (27, 28). Behavioral hardship was defined as “reporting delaying medical care due to cost in the past 12 months (dental, medical, mental health, filling prescription), needing but did not get because of the cost in the past 12 months (dental, medical, mental health, filling prescription), or skipping medication doses and taking less medication to save money (only participants who had been prescribed medications in the past 12 months were asked these questions)” (9, 27). The measure for any medical financial hardship was based on whether a respondent reported any hardship in each domain. Medical financial hardship intensity was counted based on the number of co-occurring domains. The exact wording of questions or description of recoded variables in NHIS is shown in **Supplementary Table S1**.

### 2.5 Statistical analyses

First, the prevalence of each item on the FRAIL questionnaire and the frailty level were described. The distributions of sample individual-level characteristics were also stratified by frailty level (robust *vs.* pre-frail *vs.* frail) using chi-square statistics. Then, weighted percentages were calculated for medical financial hardship domains and intensity by frailty level. Finally, multivariable logistic regression models were developed to generate odds ratios (ORs) of reporting material, psychological, and behavioral domains or any medical financial hardship by frailty level. In all multivariable regression models, the confounding effects of age, sex, race/ethnicity, education, marital status, health insurance coverage, family income level, geographic region, survey years, number of cancer diagnoses, and time since diagnosis were adjusted. Further ordinal logistic regression analyses examined the associations between hardship intensity and frailty level. Sensitivity analyses were also conducted to stratify cancer survivors by age at the time of the survey (aged <75 ≥75 years) and also by sex. The data were analyzed between 16 April and 10 May 2022. All statistical analyses used sample weights to account for the complex survey design and survey non-response of NHIS and were performed using R software (version 3.4.4). All statistical comparisons were two-sided (*α* = 0.05).

## 3 Results

**Figure 1:**
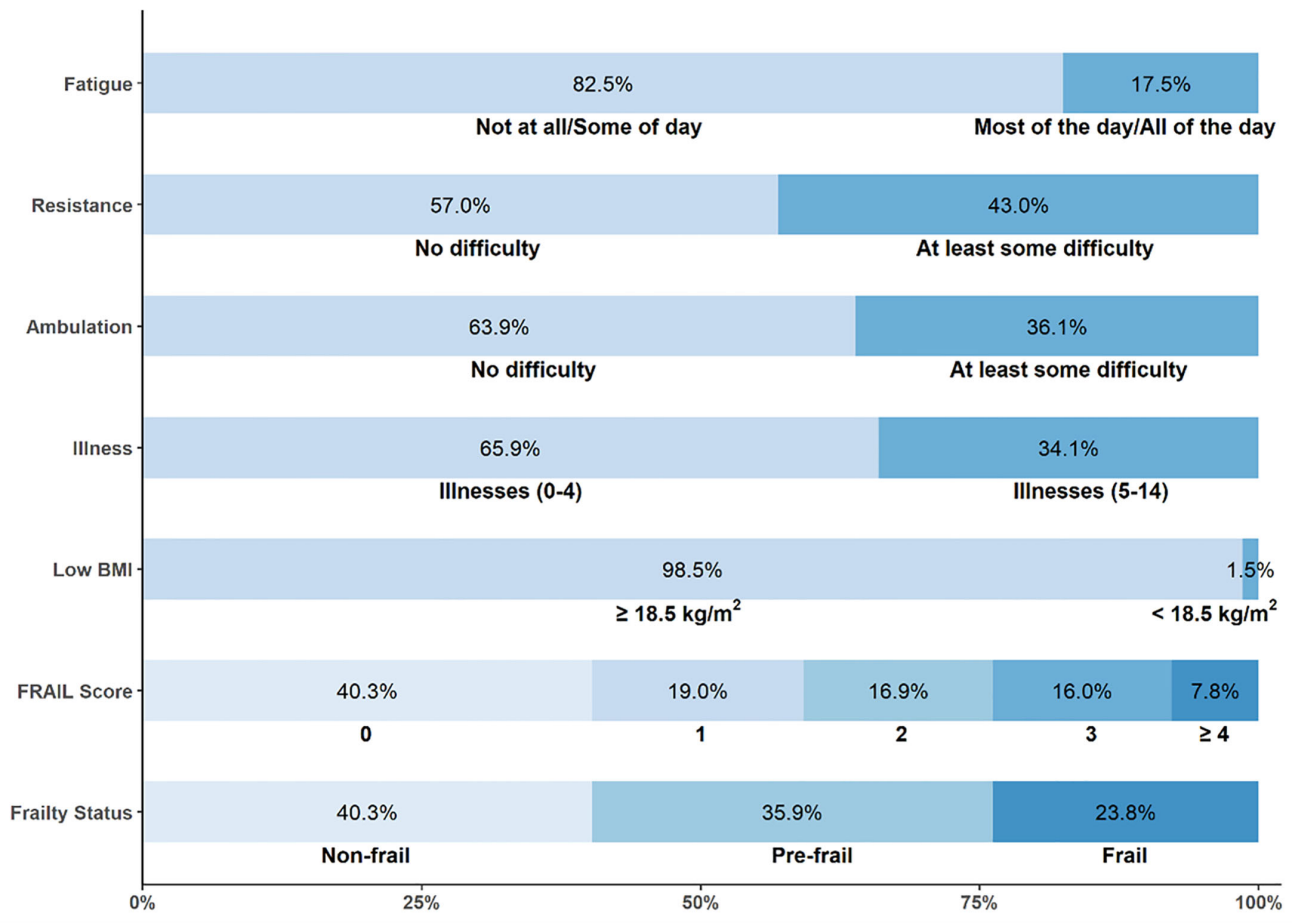
Prevalence of each FRAIL item and frailty level.

As shown in **Figure 1**, 1,583 (40.3%) older individuals with cancer were robust, 1,421 (35.9%) were pre-frail, and 915 (23.8%) were frail. Individual-level characteristics are displayed in **Table 1**. Compared with robust cancer survivors, pre-frail and frail cancer survivors were more likely to be older, female, less educated, and unmarried. They were also more likely to have a family income match 200% or less of the federal poverty level and multiple cancers.

**Table 1:** Distribution of individual-level characteristics of older adults with cancer.

Individual-level characteristics	Older adults with cancer			p
	Robust	Pre-frail	Frail	
	N = 1,583	N = 1,421	N = 915	
	%	%	%	
Age, years				<0.001
65–74	60.1	47.0	39.3	
75+	39.9	53.0	60.7	
Sex				<0.001
Male	52.7	49.2	41.5	
Female	47.3	50.8	58.5	
Race/ethnicity				0.053
Non-Hispanic white	86.4	86.8	81.7	
Non-Hispanic black	5.8	6.6	6.8	
Hispanic	4.4	4.8	6.6	
Other	3.4	1.8	4.9	
Education				<0.001
<High school	9.5	13.4	26.5	
High school graduate	22.4	29.1	27.3	
≥Some college	68.1	57.5	46.2	
Marital status				<0.001
Married	69.3	60.3	50.9	
Not married or missing^a^	30.7	39.7	49.1	
Health insurance				<0.001
Medicare and private	48.6	45.6	35.0	
Medicare and other public	11.3	15.5	21.7	
Medicare only	30.9	28.9	31.0	
Other, uninsured, or missing	9.2	10.0	12.3	
Family income level as a % of FPL				<0.001
<200%	16.9	25.0	43.9	
200%–399%	29.9	35.0	32.6	
≥400%	53.2	40.0	23.5	
Region				0.414
Northeast	17.7	18.6	17.6	
Midwest	24.3	22.8	20.2	
South	36.4	38.9	41.1	
West	21.6	19.6	21.1	
Time since cancer diagnosis				<0.001
<2 years	12.8	11.8	13.9	
≥2 years	85.0	83.9	77.4	
Missing	2.2	4.3	8.7	
Number of cancer diagnoses				<0.001
1	81.5	77.7	73.2	
≥2	18.5	22.3	26.8	

FPL, federal poverty level.

^a^ Not married includes widowed, divorced, separated, or never married.

**Figure 2:**
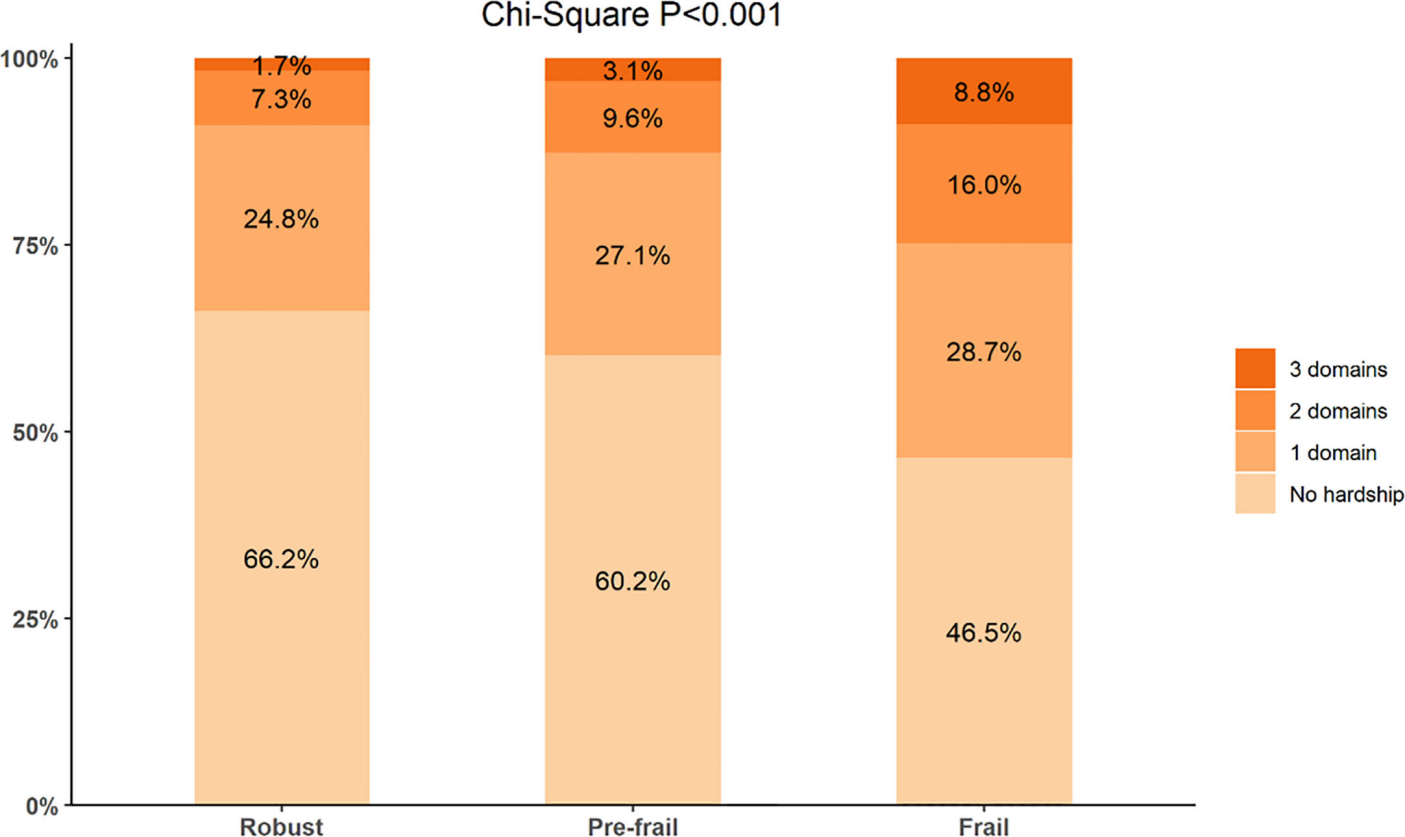
Medical financial hardship intensity and frailty level.

As presented in **Table 2**, approximately 4.6% of robust cancer survivors, 8.4% of pre-frail cancer survivors, and 18.4% of frail cancer survivors reported having problems paying medical bills. Approximately 26.0% of robust, 27.3% of pre-frail, and 38.0% of frail cancer survivors reported worrying about paying medical bills due to sickness or accidents. Cancer survivors with pre-frailty or frailty status report high rates of at least one measure of hardship in behavior compared to those with robust status (19.9% *vs.* 30.8% *vs.* 13.9%). Frail and pre-frail cancer survivors were less likely to have no hardship (46.5% *vs.* 60.2% *vs.* 66.2%, p < 0.001) and more likely to report hardship in all three domains (8.8% *vs.* 3.1% *vs.* 1.7%, p < 0.001) when compared with robust older adults with cancer (**Figure 2**).

**Table 2:** Associations of frailty level and medical financial hardship among older adults with cancer.

Financial hardship measures	Robust (Ref)	Pre-frail			Frail		
	%	%	OR (95%CI)^a^	p	%	OR (95%CI)^a^	p
Material	4.6	8.4	1.70 (1.13, 2.58)	0.012	18.4	3.19 (2.16, 4.69)	<0.001
Psychological	26.0	27.3	1.00 (0.81, 1.25)	0.976	38.0	1.47 (1.15, 1.88)	0.002
Behavioral	13.9	19.9	1.50 (1.15, 1.94)	0.003	30.8	2.45 (1.85, 3.24)	<0.001
Needed but didn’t get care	7.9	12.5	1.61 (1.17, 2.22)	0.004	22.7	2.90 (2.06, 4.06)	<0.001
Delayed medical care	11.9	16.8	1.43 (1.08, 1.90)	0.013	26.2	2.26 (1.68, 3.03)	<0.001
Other changes	2.0	3.5	1.67 (0.97, 2.87)	0.065	5.1	2.19 (1.23, 3.92)	0.008

^a^ ORs were conducted by multivariable logistic regressions. All regressions were controlled for age group, sex, race/ethnicity, education, marital status, health insurance coverage, family income, geographic region, survey years, number of cancer diagnoses, and time since diagnosis.

As shown in **Table 2**, compared with robust cancer survivors in adjusted analyses, pre-frail cancer survivors were more likely to report material domain (OR = 1.70, 95%CI: 1.13–2.58) and behavioral domain of financial hardship (OR = 1.50, 95%CI: 1.15–1.94). However, the difference in rates of psychological domain hardship was not significant between robust and pre-frail cancer survivors (26.0% *vs.* 27.3%, p = 0.976). Among the sample, frail cancer survivors were more likely than those with robust status to report issues in the material domain (OR = 3.19, 95%CI: 2.16–4.69), psychological domain (OR = 1.47, 95%CI: 1.15–1.88), and behavioral domain (ORs ranged from 2.19 to 2.90, all with p < 0.050). We also found that frail groups were similar with regard to reporting both in three domains and most measures of a behavioral domain when compared with the robust group when stratifying survivors by the COVID-19 pandemic (before *vs.* during the COVID-19 pandemic), by age (<75 *vs.* ≥75 years), and by sex (male *vs.* female) (**Supplementary Tables S2–S4**).

When comparing frailty levels (robust as the referent), we found that frailty cancer survivors had higher intensities of financial hardship (zero *vs.* at least one domain, frail: OR = 1.92, 95%CI: 1.52–2.42; zero or one domain *vs.* at least two domains, frail: OR = 2.02, 95%CI: 1.41–2.90; zero or one/two domain(s) *vs.* all three domains, frail: OR = 2.56, 95%CI: 1.40–4.67) (**Table 3**).

**Table 3:** The association of frailty and intensities of medical financial hardship among older cancer survivors.

Intensity measure level	OR^a^	95%CI	p
0 *vs.* 1, 2, 3			
Robust	Ref		
Pre-frail	1.22	(0.99, 1.49)	0.057
Frail	1.92	(1.52, 2.42)	<0.001
0, 1 *vs.* 2, 3			
Robust	Ref		
Pre-frail	1.28	(0.90, 1.82)	0.177
Frail	2.02	(1.41, 2.90)	<0.001
0, 1, 2 *vs.* 3			
Robust	Ref		
Pre-frail	1.48	(0.76, 2.87)	0.253
Frail	2.56	(1.40, 4.67)	0.002

^a^ ORs were conducted by logistic regressions. All regressions were controlled for age group, sex, race/ethnicity, education, marital status, health insurance coverage, family income, geographic region, survey years, number of cancer diagnoses, and time since diagnosis.

## 4 Discussion

### 4.1 Main findings

To our knowledge, this is the first study that focused on older adults with varying frailty levels in the nationally representative population-based database and examined its relationship with medical financial hardship in the United States. In this study, both pre-frailty and frailty statuses were associated with medical financial hardship among older cancer survivors. We found that more than one in three participants were categorized as pre-frail, and approximately one in four participants was categorized as frail. The prevalence of frailty in this study was higher (23.8% *vs.* 9.1%) than in a similar study (N = 416, aged ≥60 years) based on the Third National Health and Nutrition Examination Survey (29). Our findings that older cancer survivors with frailty status are vulnerable to the three-domain financial hardship when compared with those with robust status added knowledge on medical financial hardship during cancer survivorship. This information can also help to identify frailty conditions (previously overlooked in financial hardship research) that are very important in targeted interventions to improve older cancer survivors’ financial and health outcomes (14, 26). With the rapid growth and diversification of the older population in cancer care, these findings are novel and useful to older cancer survivorship programs given the increasing attention paid to the impact of financial status and emphasis on age-associated conditions to reduce cancer-related health disparities.

Mohile et al. demonstrated that geriatric syndromes are more common in cancer patients than in those without cancer (30, 31). Other studies also showed that older cancer survivors may be at higher risk for financial toxicity than those with other chronic conditions (4, 32). There may be significant heterogeneity in the health status of older individuals at the same chronologic age, and this study demonstrated that age alone is insufficient to inform on medical financial hardship. Moving forward, using the five items of frailty, healthcare policymakers and healthcare professionals can know more characteristics and quickly identify vulnerable, high-risk, older individuals with cancer. Preliminary data have shown that incorporating a modified FRAIL questionnaire, a simple and useful instrument for identifying geriatric risk in older cancer survivors, into understanding the financial burdens of their cancer survivorship is feasible. These findings suggest that assessment of the frailty or selected components may improve the identification of older adults at risk of medical financial hardship to better facilitate the targeting of interventions aimed at reducing the future healthcare burden. This study is considered the first step in highlighting the importance of staging the aging in patient-reported financial outcome-related studies among older adults with cancer using a FRAIL questionnaire in the United States.

There is a scarcity of nationally representative survey studies that both contain frailty and financial hardship measures and methods appropriate for older adults with cancer to build an evidence base reflecting this typical population. In this study, frailty status correlated directly with the three domains of medical financial hardship and adds important age-related concerns that are not presented by previous research. This study’s findings also provided a snapshot of the prevalence of frailty and pre-frailty among older adults with cancer survivors in the United States in the 2020s. Frailty groups deserve special attention, and if this problem is not addressed, growing medical financial hardship may also be associated with widening cancer disparities and worsened outcomes. Older cancer patients are often given complex information about the risks and benefits of cancer treatment, but their age-related problems and outcomes are not usually mentioned (33, 34). Incorporating frailty screening into the medical decision-making process for older cancer patients may help to find aging conditions that are often overlooked in routine oncology care but are important for these populations (35).

Given greater aging and rapid development of frailty, the experience of medical financial hardship is likely to increase and may exacerbate cancer-related health disparities. Older cancer survivors are more likely to have reduced resources to pay for medical care, thereby increasing the financial impact of cancer. Cancer survivors with frailty status have been shown to have more material, psychological, and behavioral financial hardships. Poorer quality of life and overall wellbeing, increased stress, restricted choices associated with limited resources, and decreased healthcare adherence are among the potential hypothesized mechanisms for the association between frailty and financial hardship (27). The mechanism for this increased psychological financial hardship is not entirely clear. Psychological domains were usually measured as any psychological, emotional, and social impact experienced by cancer survivors because of financial hardship. Although specific pathways are unknown, previous research (10, 27) showed that the feeling of distress because of costs of healthcare and concern about wages/income meeting expenses related to costs of healthcare may cause a shift in the attention of older cancer survivors away from material conditions to focus on psychological effects. It is likely that cancer survivors who are frail or pre-frail may have more financial distress and worry about medical costs.

### 4.2 Clinical implications

Given that cancer is often a long-term and age-related illness, staging the aging in cancer survivors should be considered as important as staging the cancer stage. As a large proportion of older cancer patients experience frailty status, which negatively impacts their experience of medical financial hardship, early frailty screening and preventive strategies are necessary to reduce financial hardship through decision-making and pretreatment optimization in the growing geriatric oncology population. Therefore, frailty assessments could be useful for stratifying aging status and identifying older adults with cancer who experienced more medical financial hardship, as well as for reducing medical costs by improving frailty status. In this observational study, the summarized evidence supports the integration of FRAIL metrics from NHIS items to understand the complex frailty level among older cancer survivors. A previous study showed that because this tool can be self-administered and does not require a face-to-face physical examination, it can be an efficient and cost-effective way to screen large numbers of people for frailty (26). Early frailty screening can allow oncologists to discriminate robust individuals from frailty individuals from the heterogeneous elderly patient population. If medical resources are available, the management of frailty survivors should be multidisciplinary. If not, they should be offered at least cautious medical attention to reduce medical financial hardship and improve their quality of life.

This study’s findings warrant future research to create frailty interventions that may need to be implemented to help those with robust or pre-frailty status avoid frailty from ever developing. A previous study showed that successful exercise, physical activity, pharmaceutical trials, and dietary interventions can prevent or remediate frailty in older adults with cancer (18). In addition, non-oncologic aging interventions to better understand the value of frailty may improve survivors’ health-related quality of life and satisfaction with medical experience, as well as mitigate their medical financial hardship; psychological, mobility, comorbidity, medication management, and nutritional interventions are recommended for individualized management strategies to optimize care for the individual with pre-frailty or frailty status (36). Recently, a nationwide trial study found that a geriatric assessment intervention can improve patient–oncologist communication about aging-related problems in robust, pre-frail, and frail older adults with advanced cancer (37).

### 4.3 Strengths and limitations

This study’s strengths include the latest nationally representative older cancer sample and well-designed measures to quantify frailty and medical financial hardship. We provide a novel approach to measuring FRAIL to localize older individuals at high risk. Although these five questions of frailty are not validated, it is believed that the quantified results can elucidate frailty. These strengths facilitate the ability to provide national estimates of frailty prevalence among older cancer survivorship and identify frailty level as a risk factor for medical financial hardship for the first time.

Consistent with other national survey studies (28, 38), this study also has several limitations, such as cross-sectional study design, the possibility of reporting errors due to self-reporting, and relatively low response rates. Due to the rotating questions of availability from NHIS data, the item Fatigue from the NIHS questions was measured differently in 2019 and 2020, so the extrapolation of the FRAIL instrument may be limited. This study also lacks data on the stage of cancer and the details of cancer treatment, as well as the differences with regard to the drivers of financial hardship among older patients with different cancer trajectories, which should be examined because these drivers may vary by the stage of disease or by treatment modalities. In addition, because the age and age of cancer diagnosis answers were both top-coded 85 by the NHIS, researchers were unable to calculate more details of time since cancer diagnosis for some of the oldest (≥85 years) samples. Therefore, this study was unable to conclude that those samples would provide similar results.

## 5 Conclusions

In summary, this study found that both pre-frailty and frailty statuses are common in older adults with cancer and that frailty status is associated with multiple domains of financial hardship. This knowledge will help ongoing research about important age-related concerns among older cancer survivorship care. Efforts to target interventions should address geriatric vulnerabilities during the medical decision-making process and cancer survivorship.

## Data availability statement

Publicly available datasets were analyzed in this study. This data can be found here: http://www.cdc.gov/nchs/nhis.htm.

## Ethics statement

The National Center for Health Statistics ethics review board approved all the NHIS protocols, and all survey participants provided informed consent to participate in NHIS. Therefore, no informed consent was required.

## Author contributions

Conceptualization: JL and MS. Methodology: JL and MS. Formal analysis: JL. Data curation: JL. Writing—original draft preparation: MS. Writing—review and editing: JL, LL, JZ, SZ, and NY. Visualization: JL and MS. Supervision: NY. All authors contributed to the article and approved the submitted version.
