# Supplementary material for: Frailty and medical financial hardship among older adults with cancer in the United States
Source: Front Oncol. 2023 Jun 29;13:1202575. doi: 10.3389/fonc.2023.1202575 (PMC10344591; doi:10.3389/fonc.2023.1202575)
Supplement: Supplementary file 1 [file DataSheet_1.docx]

**Supplementary Table S1** Measures of medical financial hardship, and FRAIL questionnaire in NHIS

| **Medical financial hardship measures** | | | **NHIS question** | **Years of availability** |
| --- | --- | --- | --- | --- |
| Material | | Problems paying medical bills | In the past 12 months did you/anyone in the family have problems paying or were unable to pay any medical bills? | 2019-2020 |
|  |  | Unable to pay medical bills | Do you/Does anyone in your family currently have any medical bills that you are unable to pay at all? |  |
| Psychological | | Get sick or have accident, worry about paying medical bills | If you get sick or have an accident, how worried are you that you will be able to pay your medical bills? Are you very worried, somewhat worried, or not at all worried? |  |
| Behavioral | Delayed medical care due to cost | Dental care | During the past 12 months, have you DELAYED getting dental care because of the cost? |  |
|  |  | Medical care | During the past 12 months, have you DELAYED getting medical care because of the cost? |  |
|  |  | Mental health care | During the past 12 months, have you DELAYED getting counseling or therapy from a mental health professional because of the cost? |  |
|  |  | Filling prescription | During the past 12 months, were any of the following true for you? You DELAYED filling a prescription to save money. |  |
|  | Needed but didn't get because of the cost | Dental care | During the past 12 months, was there any time when you needed dental care, but DID NOT GET IT because of the cost? |  |
|  |  | Medical care | During the past 12 months, was there any time when you needed medical care, but DID NOT GET IT because of the cost? |  |
|  |  | Counseling/therapy | During the past 12 months, was there any time when you needed prescription medication, but DID NOT GET IT because of the cost? |  |
|  |  | Prescription medication | During the past 12 months, was there any time when you needed counseling or therapy from a mental health professional, but DID NOT GET IT because of the cost? |  |
|  | Other changes in prescription medication use | Skipped medication doses to save money | During the past 12 months, were any of the following true for you? ...You skipped medication doses to save money. |  |
|  |  | Took less medication to save money | During the past 12 months, were any of the following true for you? ...You took less medication to save money. |  |
| Frailty measures | | **NHIS question or variable description** | | **Years of availability** |
| Fatigue | | Over the last two weeks, how often have you been bothered by feeling tired or having little energy? Would you say not at all, several days, more than half the days, or nearly every day? | | 2019 |
|  |  | Thinking about the last time you felt very tired or exhausted, how long did it last? Would you say some of the day, most of the day, or all of the day? | | 2020 |
| Resistance | | Do you use any equipment or receive help for getting around? | | 2019-2020 |
|  |  | Do you have difficulty walking up or down 12 steps? | |  |
| Ambulation | | Do you use any equipment or receive help for getting around? | |  |
|  |  | Do you have difficulty walking a third of a mile on level ground, that would be the length of five football fields or five city blocks? | |  |
| Illness | | Have you EVER been told by a doctor or other health professional that you had [Illness]? (hypertension, high cholesterol, coronary heart disease, angina, heart attack, stroke, asthma, cancer, diabetes, chronic obstructive pulmonary disease, arthritis, and anxiety disorder, depression, dementia) | |  |
| Low Body Mass Index | | Categorical body mass index, public use | |  |

**Supplementary Table S2** Associations of Frailty Level and Medical Financial Hardship among Older Cancer Survivors, by Before and During COVID-19 Pandemic

| **Financial hardship measures** | **Frailty level**^a^ | **Before COVID-19** | **During COVID-19** | **Wald test** |
| --- | --- | --- | --- | --- |
|  |  | **OR (95%CI)^b^** | **OR (95%CI)^b^** | **P** |
| Material | Pre-frail | 1.71 (1.06, 2.77) | 1.58 (0.75, 3.35) | 0.846 |
|  | Frail | 3.05 (1.91, 4.85) | 3.14 (1.57, 6.25) | 0.926 |
| Psychological | Pre-frail | 1.19 (0.92, 1.55) | 0.86 (0.62, 1.20) | 0.113 |
|  | Frail | 1.79 (1.36, 2.36) | 1.26 (0.86, 1.86) | 0.061 |
| Behavioral | Pre-frail | 1.23 (0.91, 1.67) | 1.75 (1.16, 2.63) | 0.170 |
|  | Frail | 1.97 (1.41, 2.74) | 3.01 (1.93, 4.70) | 0.312 |
| Needed but didn't get care | Pre-frail | 1.40 (0.97, 2.03) | 1.87 (1.11, 3.16) | 0.325 |
|  | Frail | 2.73 (1.86, 4.01) | 3.14 (1.78, 5.54) | 0.776 |
| Delayed medical care | Pre-frail | 1.20 (0.86, 1.67) | 1.64 (1.05, 2.56) | 0.255 |
|  | Frail | 1.88 (1.31, 2.72) | 2.66 (1.67, 4.26) | 0.512 |
| Other changes | Pre-frail | 1.52 (0.75, 3.10) | 1.72 (0.71, 4.12) | 0.962 |
|  | Frail | 2.94 (1.45, 5.94) | 1.55 (0.55, 4.39) | 0.150 |

^a^ The comparison groups were individuals with robust.

^b^ ORs were conducted by multivariable logistic regressions. All regressions controlled for age group, sex, race/ethnicity, education, marital status, health insurance coverage, and family income, geographic region, number of cancer diagnosis, and time since diagnosis.

**Supplementary Table S3** Associations of Frailty Level and Medical Financial Hardship among Older Cancer Survivors, by Age (< and ≥ 75 Years)

| **Financial hardship measures** | **Frailty level^a^** | **< 75 Years** | **≥ 75 Years** | **Wald test** |
| --- | --- | --- | --- | --- |
|  |  | **OR (95%CI)^b^** | **OR (95%CI)^b^** | **P** |
| Material | Pre-frail | 2.13(1.26, 3.59) | 1.34(0.68, 2.65) | 0.274 |
|  | Frail | 4.59(2.75, 7.66) | 2.31(1.26, 4.21) | 0.096 |
| Psychological | Pre-frail | 1.04(0.77, 1.40) | 0.97(0.69, 1.35) | 0.598 |
|  | Frail | 1.44(1.02, 2.03) | 1.50(1.05, 2.15) | 0.915 |
| Behavioral | Pre-frail | 1.82(1.32, 2.50) | 1.19(0.78, 1.80) | 0.081 |
|  | Frail | 2.16(1.49, 3.14) | 2.78(1.82, 4.23) | 0.508 |
| Needed but didn't get care | Pre-frail | 1.88(1.26, 2.81) | 1.30(0.79, 2.13) | 0.208 |
|  | Frail | 2.70(1.76, 4.15) | 3.13(1.87, 5.24) | 0.962 |
| Delayed medical care | Pre-frail | 1.81(1.27, 2.58) | 1.03(0.65, 1.63) | 0.046 |
|  | Frail | 2.02(1.35, 3.02) | 2.45(1.56, 3.84) | 0.586 |
| Other changes | Pre-frail | 1.73(0.89, 3.34) | 1.23(0.44, 3.40) | 0.511 |
|  | Frail | 2.43(1.18, 5.03) | 1.49(0.51, 4.38) | 0.288 |

^a^ The comparison groups were individuals with robust.

^b^ ORs were conducted by multivariable logistic regressions. All regressions controlled for sex, race/ethnicity, education, marital status, health insurance coverage, and family income, geographic region, survey years, number of cancer diagnosis, and time since diagnosis.

**Supplementary Table S4** Associations of Frailty Level and Medical Financial Hardship among Older Cancer Survivors, by sex

| **Financial hardship measures** | **Frailty level^a^** | **Male** | **Female** | **Wald test** |
| --- | --- | --- | --- | --- |
|  |  | **OR (95%CI)^b^** | **OR (95%CI)^b^** | **P** |
| Material | Pre-frail | 1.86(1.04, 3.34) | 1.59(0.85, 2.98) | 0.755 |
|  | Frail | 2.72(1.50, 4.94) | 3.45(1.94, 6.13) | 0.478 |
| Psychological | Pre-frail | 0.99(0.72, 1.35) | 1.06(0.80, 1.40) | 0.718 |
|  | Frail | 1.52(1.04, 2.20) | 1.51(1.11, 2.05) | 0.731 |
| Behavioral | Pre-frail | 1.57(1.03, 2.39) | 1.51(1.09, 2.08) | 0.960 |
|  | Frail | 1.97(1.27, 3.06) | 2.86(2.04, 4.01) | 0.197 |
| Needed but didn't get care | Pre-frail | 1.63(0.99, 2.69) | 1.68(1.15, 2.44) | 0.977 |
|  | Frail | 2.51(1.48, 4.26) | 3.31(2.20, 4.98) | 0.722 |
| Delayed medical care | Pre-frail | 1.62(1.02, 2.57) | 1.37(0.97, 1.92) | 0.742 |
|  | Frail | 1.76(1.09, 2.83) | 2.65(1.86, 3.77) | 0.107 |
| Other changes | Pre-frail | 1.31(0.56, 3.06) | 2.44(1.26, 4.72) | 0.301 |
|  | Frail | 0.55(0.18, 1.68) | 5.01(2.48, 10.10) | 0.005 |

^a^ The comparison groups were individuals with robust.

^b^ ORs were conducted by multivariable logistic regressions. All regressions controlled for age group, race/ethnicity, education, marital status, health insurance coverage, family income, geographic region, number of cancer diagnosis, and time since diagnosis.


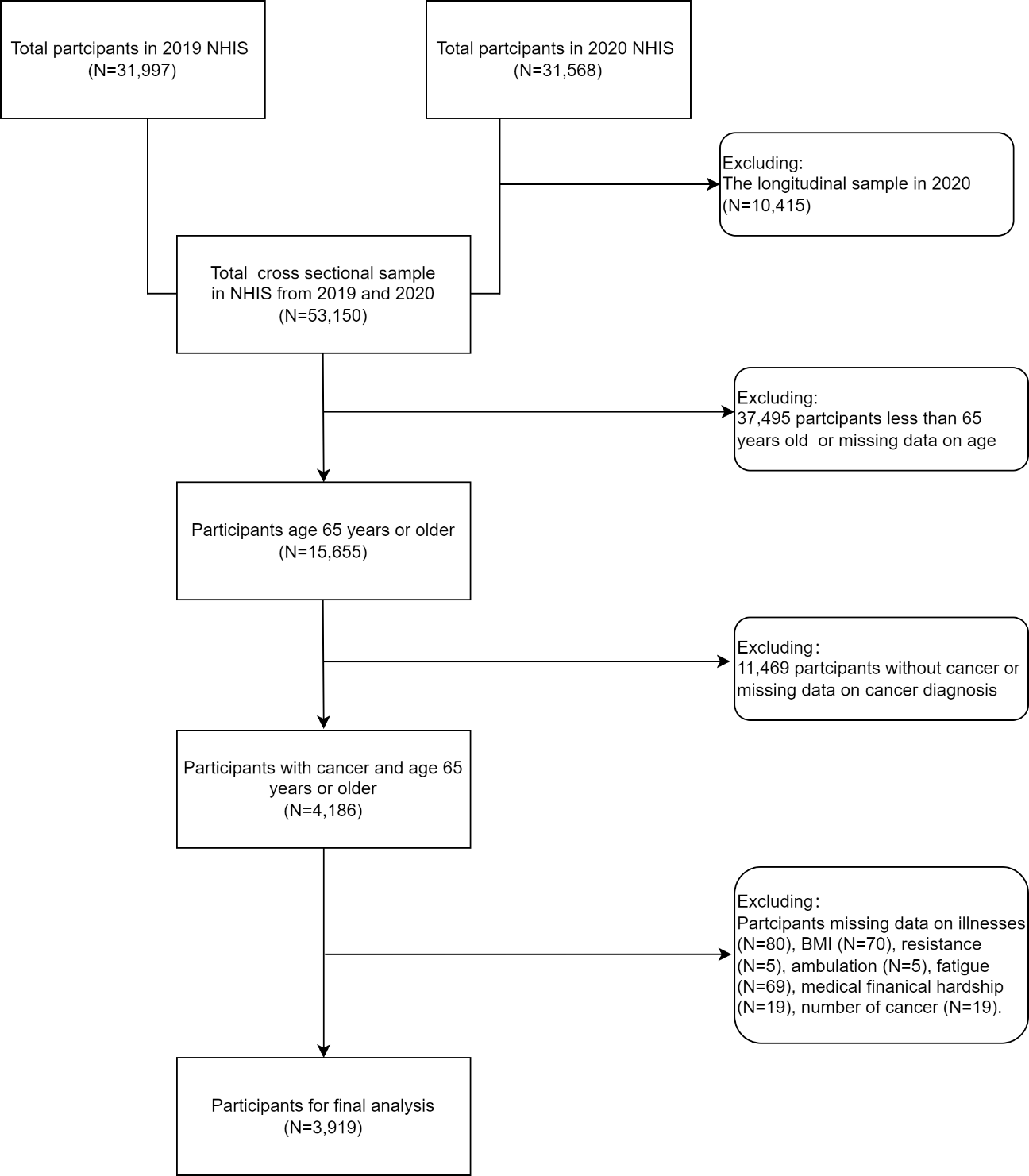


**Supplementary Figure S1.** Flowchart showing the selection of the older cancer survivors enrolled in the NHIS.
